# Understanding the Entanglement: Neutrophil Extracellular Traps (NETs) in Cystic Fibrosis

**DOI:** 10.3389/fcimb.2017.00104

**Published:** 2017-04-06

**Authors:** Saira R. Martínez-Alemán, Lizbeth Campos-García, José P. Palma-Nicolas, Romel Hernández-Bello, Gloria M. González, Alejandro Sánchez-González

**Affiliations:** Departamento de Microbiología, Facultad de Medicina, Universidad Autónoma de Nuevo LeónMonterrey, Mexico

**Keywords:** neutrophil, NETs, cystic fibrosis, CFTR, *Pseudomonas aeruginosa*, *Staphylococcus aureus*, *Burkholderia cepacia*

## Abstract

Cystic fibrosis (CF) is an autosomal recessive disorder caused by mutations in the gene that codes for the CF trans-membrane conductance regulator. These mutations result in abnormal secretions viscous airways of the lungs, favoring pulmonary infection and inflammation in the middle of neutrophil recruitment. Recently it was described that neutrophils can contribute with disease pathology by extruding large amounts of nuclear material through a mechanism of cell death known as Neutrophil Extracellular Traps (NETs) into the airways of patients with CF. Additionally, NETs production can contribute to airway colonization with bacteria, since they are the microorganisms most frequently found in these patients. In this review, we will discuss the implication of individual or mixed bacterial infections that most often colonize the lung of patients with CF, and the NETs role on the disease.

## Introduction

Cystic Fibrosis (CF) is a lung disease characterized by chronic inflammation of the airways associated with bacterial colonization. Disease occurs by mutations that disrupt the Cystic Fibrosis Transmembrane Regulator (CFTR) gene, a plasma membrane channel that regulates the balance of bicarbonate and chloride secretions across the epithelial layer of the airways. Patients with CF have an elevated presence of thick mucus, DNA and proteins, complexed with bacteria that obstruct airflow due to an inability of the epithelial cilia to beat and mediate mechanical clearance of these structures. More recently, some studies showed that the extracellular DNA levels correlate with neutrophil counts and can be used as a guide of inflammation and lung disease severity (Kirchner et al., 1996; Ratjen et al., 2005).

Neutrophil Extracellular Traps (NETs) release was recently related to the extracellular DNA found in patients with CF in which their presence can exacerbate the disease. The excessive formation of NETs promotes that the mucus in patient's alveoli becomes thicker and sticky, allowing the colonization of bacteria such as *Haemophilus influenzae* and *Staphylococcus aureus*, and significantly *Pseudomonas aeruginosa* (Manzenreiter et al., 2012). Such colonization promotes infiltration of neutrophils that undergo NETosis increasing sputum viscosity and consequently lowering the patient's respiratory capacity. In line with this, a treatment option for patients with CF is the administration of recombinant human DNase to disrupt NETs and so liquefy the sputum and facilitate mucociliary clearance (Papayannopoulos et al., 2011).

## The neutrophil and its characteristics

Neutrophils are the most abundant white blood cells in circulation and have an important role in the immune response (Amulic et al., 2012). They possess a segmented nucleus and in their cytoplasm, multiple granules and secretory vesicles can be found. These granules have been subdivided into three classes: azurophilic, specific and gelatinase (Kolaczkowska and Kubes, 2013). Neutrophils also produce many peptides and proteins that directly or indirectly kill microbes divided into three types of antimicrobials: cationic peptides (i.e., defensins and cathelicidins); proteolytic enzymes [i.e., lysozyme, myeloperoxidase and neutrophil elastase (NE)] and, metal chelating proteins (i.e., lactoferrin and calprotectin; Borregaard and Cowland, 1997).

When a pathogen is detected, neutrophils leave the blood vessels and are recruited to the site of infection following a chemotactic gradient produced by microbial and endogenous signals (Papayannopoulos and Zychlinsky, 2009; Brinkmann and Zychlinsky, 2012). In the inflammatory site, these cells employ intracellular and extracellular strategies to contain and eliminate the infection (Kolaczkowska and Kubes, 2013). Intracellularly, eliminate pathogens very effectively by phagocytosis and degradation of the cargo in the lizosome, whereas, extracellular mechanims include degranulation where the lytic granules contained in vacuoles are expelled, production of reactive oxygen species that can affect directly the pathogen proteins and DNA and formation of extracellulat traps which can restrict dissemination at the same time that promote pathogen killing (Brinkmann et al., 2004; Papayannopoulos and Zychlinsky, 2009).

## Neutrophil extracellular traps

NETs formation was first described in 2004 and although plenty of information has arised still many aspects of its regulation are unknown (Brinkmann and Zychlinsky, 2012). To release NETs, the activated neutrophil undergoes dramatic morphological changes mediated by signaling pathways that differ depending on the stimulus. It has been found that chemical inductors such as PMA promote “suicidal NETs” formation in which neutrophils extrude their DNA and die, a process that takes of 1.5–2 h to ocurr (Brinkmann and Zychlinsky, 2012; Zawrotniak and Rapala-Kozik, 2013) on the other hand, pathogen inducers promote “vital NETs” formation in matters of minutes (0.5–1 h) in which the cells extrude their DNA but still can migrate and kill pathogens by phagocytosis (Yipp and Kubes, 2016).

In the initial steps of NETs formation, minutes after activation, the neutrophil flattens and adheres firmly to a substrate, such adhesion is mediated by integrin receptors (Neeli et al., 2008). When “suicidal NETs” are induced neutrophil binding to its substrate promotes the activation of the NADPH oxidase complex, when is assembled in the fagosomal cell membrane and reduces the molecular oxygen superoxide anion by electron transfer. Superoxide dismutase (SOD) converts superoxide anion into hydrogen peroxide (H_2_O_2_), which acts in turn as a substrate for the enzyme myeloperoxidase (MPO), and also reacts with H_2_O_2_ to generate hypochlorous acid (Brinkmann and Zychlinsky, 2012). In contrast, ROS formation is not escential in “vital NETs” since in this a NADPH oxidase independent mechanism has been described to some pathogens (Rochael et al., 2015; Yipp and Kubes, 2016). In a subsequent step, the nucleus loses its lobed shape and the chromatin is decondensed by action of NE and MPO enzymes, that migrate to the nucleus where they exert their proteolytic function on histones (Fuchs et al., 2007; Papayannopoulos et al., 2010). Concomitantly, chromatin decondensation can be increased due to epigenetic mechanisms on histones, as it was found that histones H3 and H4 can be modified by a reaction catalyzed by arginine peptidyl deaminase 4 (PAD4) that converts arginine residues to citrulline (Wang et al., 2009; Leshner et al., 2012). Later, the nuclear envelope disintegrates, nucleoplasm and cytoplasm are mixed forming a homogeneous mass, and finally, rupture of the cell membrane promotes that the cellular contents are expelled into the extracellular space (Fuchs et al., 2007; Brinkmann and Zychlinsky, 2012).

## The function of NETs

NETs likely evolved to restrict infections due to its ability to entrap, prevent the spread and exterminate microorganisms (Brinkmann and Zychlinsky, 2012), as they can catch almost all types of pathogens as its presence has been demonstrated in response against gram-positive and gram-negative bacterial infections, yeasts, viruses and protozoan parasites (Lu et al., 2012). Capture within the fibers of DNA prevents propagation of microorganisms on the body and facilitates a higher concentration of antimicrobial factors at the site of infection (Brinkmann et al., 2004). This capture occurs through charge interactions between cell surface components of the pathogen and NETs and its antimicrobial functions are exerted by the granular proteins, primarily by NE, histone and MPO, but also by the action of calgranulin, proteinase 3, lactoferrin, calprotectin, and antimicrobial peptides (AMP) such as defensins or cathelicidin LL-37 (Urban et al., 2009).

In addition to the antimicrobial function of NETs, its ineffective elimination or excessive presence can cause pathological effects. NETs formation has been observed during chronic inflammatory diseases (atherosclerosis), autoimmune diseases such as systemic lupus erythematosus (SLE), in various forms of vasculitis, thrombosis, as well as pulmonary diseases such as CF (Kolaczkowska and Kubes, 2013).

## Cystic fibrosis and its relationship with NETs

CF is a disease produced by mutations that disrupt the CFTR gene (Table 1); the effect of these mutations is the production of abnormally viscous secretions in the airways of the lungs causing obstructions, inflammation and tissue destruction (Cutting, 2015). On the other hand, an important role on the immunopathology of the disease has been given to immune cells. Particularly, considerable participation of neutrophils has been proposed, as these cells have an increased influx to the lungs of the patients in response to bacterial colonization (Gray et al., 2015), but present a reduced ability to remove microbes due to an ineffective respiratory burst (Painter et al., 2008) and induce an exacerbated local tissue damage because of uncontrolled degranulation (Rogan et al., 2004; Sagel et al., 2004; Bergsson et al., 2009). Recently, NETs formation has drawn attention as it has been found that once free DNA is present in high quantity of in the sputum, patients present diminished lung function when compared with patients that have mild disease, indicating that the airway obstruction is due to the accumulation of NETs-DNA (Papayannopoulos et al., 2011; Dubois et al., 2012; Dwyer et al., 2014; Marcos et al., 2015). Also, it has been reported that components of NETs (i.e., mieloperoxidase, neutrophil elastase and histones) can induce destruction of epithelial, endothelial and connective tissue worsening the lung pathology (Klebanoff et al., 1993; Manzenreiter et al., 2012; Saffarzadeh et al., 2012). The importance of NETs in this disease is underscored by observations describing that elimination of free DNA from patient's airways constitute an important treatment option for combating CF since administration of recombinant human DNase, leads to a significate improvement on health (Rahman and Gadjeva, 2014).

**Table 1:** **Classes of CFTR mutations that cause cystic fibrosis**.

**Class**	**Defect**	**Example**	**References**
I	About half of the CFTR mutations are expected to prevent proper synthesis of full-length, normal CFTR polypeptide because of nonsense, frameshift, or aberrant splicing of mRNA.	G542X	Rowe et al., 2005
II	The defective protein retains substantial chloride-channel function in cell-free lipid membranes. When synthesized by the normal cellular machinery, however, the protein is rapidly recognized as misfolded and is degraded shortly after synthesis, before it can reach its crucial site of action at the cell surface.	ΔF508 N1303K G85E G91R	O'Sullivan and Freedman, 2009
III	It encodes properly processed, full-length CFTR protein that lacks normal ion-channel activity	G551D G551S G1244E G1255P G1349D	Rowe et al., 2005
IV	This mutation exhibits only partial CFTR ion-channel activity, a feature that probably explains a less severe pulmonary phenotype.	A455E R117H R334W R347P	Gan et al., 1995
V	It includes promoter mutations that reduce transcription, nucleotide alterations that promote alternative splicing of the CFTR transcript, and amino acid substitutions that cause inefficient protein maturation.	P574H A455E	Welsh and Smith, 1993

## NETs formation by microorganisms of cystic fibrosis

In the lungs, the first point of contact for contaminants and microorganisms is the respiratory tract (Cullen and McClean, 2015). Sophisticated host defense mechanisms in the mucosa of the lungs, especially in healthy individuals, as well as the presence of cilia and mucus epithelial surface prevent infections trapping and removing particles and microorganisms of the healthy lung, even though the microorganisms are constantly inhaled (Eisele and Anderson, 2011). CF is characterized by airway inflammation, increased viscous mucus production and reduced mucociliary clearance, favoring chronic infections that contribute to a rapid decline in lung function and health (Cullen and McClean, 2015).

The alterations in the lung surface provides a suitable environment for the growth of various pathogens, being the most represented bacteria and fungi; among which, *Pseudomonas aeruginosa* constitutes one of the most prevalent pathogens that colonizes the lungs of CF patients (Cullen and McClean, 2015). Elimination of this bacteria results very difficult for the immune system since it is known that when infects the patient's lungs, this pathogen can migrate to areas with low oxygen concentrations where only few immune cells can exert its function (Young et al., 2011). Among these cells, the neutrophils constitute the primary defense line but *P. aeruginosa* has developed strategies to evade their destruction, as it has been shown that biofilm formation promotes excessive production of alginate that allows bacterial evasion to neutrophil phagocytosis and degranulation (Mulcahy et al., 2008). Additionally, *P. aeruginosa* has obtained diverse mechanisms to evade the effects of NETs, among these it is know that they can counteract the capacity of these structures to chelate divalent metal cations by overexpression of genes controlled by the two component systems PhoPQ and PmrAB that sense Mg^2+^ limitation and at the same time encodes mechanisms to effectively obtain the ion (Mulcahy et al., 2008; Johnson et al., 2012, 2013). Additionally, the bacteria is capable of regulating genes that allow them to tolerate the toxicity caused by the extracellular DNA (Halverson et al., 2015).

Respiratory tract colonization of CF patients by *Staphylococcus aureus* has drawn increasing attention, as it has been found that they suffer a more aggressive form of the disease. Possible explanations to this are not well understood, but the effect has been attributed to the high production of bacterial toxins that can induce strong damage to the patient's airways and to the resistance that this bacterium has against the immune mechanisms which include phagocytosis and degranulation evasion through production of capsule (Menestrina et al., 2003; Voyich et al., 2005). The role of NETosis in the response against *S. aureus* in CF is still not completely known but now is suggested that these structures can be more harmful that beneficial for patient's health. It has been reported that toxins produced by this bacterium can induce different types of cell death depending on the concentration including NETs (Genestier et al., 2005), but their antimicrobial effect could be limited due to a production a DNAse by the bacteria (Pilsczek et al., 2010) and moreover could contribute to tissue damage by increasing the local concentration of cytotoxic molecules. Additionally, experiments *in vitro* have shown that neutrophils infected with *S. aureus* can induce NETs with a faster kinetic resembling “vital NETs” formation (Pilsczek et al., 2010), and other group demonstrated that these structures are modified in a way to become cytotoxic for macrophages allowing the establishment of chronic infections and increased tissue damage (Thammavongsa et al., 2013).

Other groups of bacteria that have been found colonizing the respiratory tract of CF patients include *Haemophilus influenza, Stenotrophomonas maltophilia, Achromobacter xyloxidans*, genus *Pandoraea* and *Streptococci* (LiPuma, 2010; Callaghan and McClean, 2012). However, it has not been reported the formation of NETs by any of these bacteria in CF and its role on the disease still remains to be explored. A growing concern among clinicians is the observation of adult patient's lungs colonization with *Burkholderia cepacia* since it has been related to decreased respiratory function and worsening of the disease, the mechanisms behind this are not understood although it has been proposed a possible association with previously colonizing bacteria (*Pseudomonas aeruginosa* and/or *Staphylococcus aureus*) to form mixed clusters of pathogens.

NETs and bacterial biofilms can be found in high amounts in CF patient's lung, the possible relationship of these structures induced by bacteria colonizing the alveoli of CF patients is an interesting aspect to study, as it could be possible that the pathogen promotes biofilm and NET formation to use these structures as a niche to stablish the infection and persist at the cost of a detriment in patient's health. The idea results attractive as some groups have shown that in otitis media and supragingival infections some surface bacterial proteins determinants for biofilm formation can also attract neutrophils to the site of infection to induce NETs and at the same time contribute to avoid their elimination by the induced traps and phagocytic killing by other incoming cells (Juneau et al., 2011; Hirschfeld et al., 2015).

**Figure 1:**
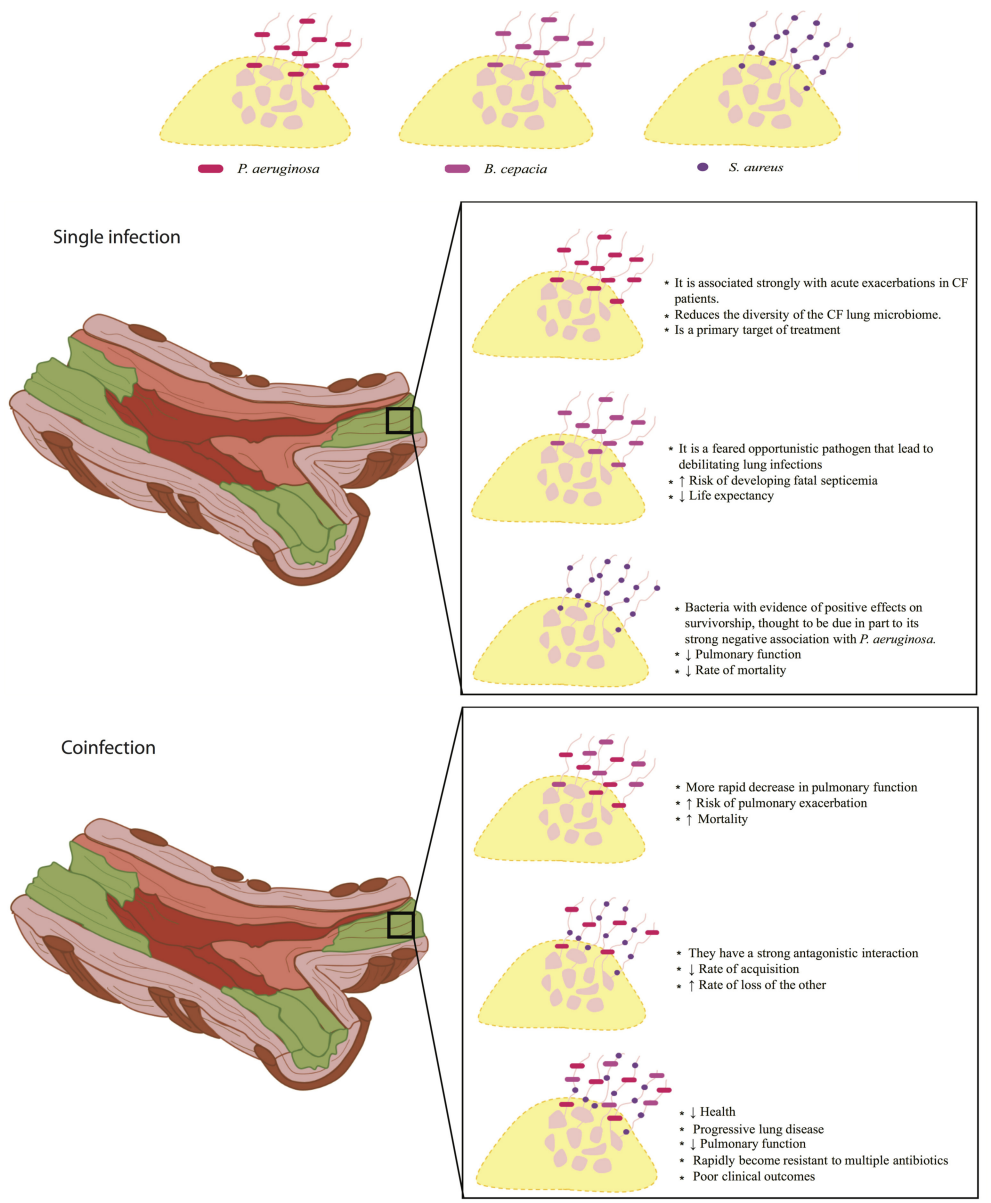
**NET formation in the lung surface of cystic fibrosis patients**. Implications on disease prognosis (text box) in bacterial single infections (middle panel) and coinfections (lower panel) are shown. NET formation can constitute a scaffold for the establishment of simple infections or coinfections that can directly influence the elimination of pathogens that have direct repercussions on patient's health. Upper panel, within single infections, *P. aeruginosa* is the main bacterium that colonizes the lungs of patients with cystic fibrosis, and is associated with decreased microbiome as compared to healthy individuals. Patients colonized with *B. cepacia* are at higher risk of developing septicemia by decreasing their life expectancy. On the other hand, *S. aureus* is less aggressive compared to *P. aeruginosa* and *B. cepacia*, despite this, it can decrease lung function and increase the mortality rate. Bottom panel, mixed infections caused by *P. aeruginosa* and *B. cepacia* may become the most aggressive, due to a rapid loss of lung function, increasing the risk of pulmonary exacerbation and mortality. Coinfections between *P. aeruginosa* and *S. aureus*, have a very antagonistic interaction in which they compete for their establishment by decreasing the rate of acquisition and the rate of loss of another, such battle promote increased lung damage and rapid worsening of patient's health. On the other hand, coinfections between *P. aeruginosa, S. aureus*, and *B. cepacia* causes progressive lung disease and decreased lung function severely compromising patients' health.

Figure 1 summarizes the information about the effect of mixed or single infections on the CF. *P. aeruginosa* is considered the main bacterium that affects people with CF, causing chronic lung infections, which leads to high rates of morbidity and mortality, and once the bacteria is established, it is difficult to eradicate it (Winstanley et al., 2016). On the other hand, patients colonized in a chronic manner with *B. cepacia* present a worse prognosis of the disease, as the use of antibiotics becomes more frequent they also have a greater deterioration of lung function and the rate of mortality is higher compared to that patients colonized by only *P. aeruginosa* (Gilligan, 2014; Folescu et al., 2015). It has been reported that *B. cepacia* and *P. aeruginosa* can form mixed biofilms in the lungs of people with CF, since, *P. aeruginosa* through its extracellular products can increase the attachment of *B. cepacia* by modifying the lung epithelial cells on its surface; however, the same does not occur in patients previously colonized with *B. cepacia* (Saiman et al., 1990). Coinfection between the two bacteria results in a rapid decline in lung function and a high mortality rate (Folescu et al., 2015). In CF the simple colonization by *S. aureus* is generally considered of better prognosis than those colonized with *P. aeruginosa*; but in some cases, the presence of small-colony variants (SCV) of *S. aureus* is associated with the most advanced lung disease in CF, and the phenotype of this bacterium in a coinfection with *P. aeruginosa* causes disease worsening (Besier et al., 2007; Hubert et al., 2013). In addition, some groups have found that host-pathogen interactions can affect the environment favoring pulmonary disease (Filkins and O'Toole, 2015) and that high levels of inflammation as well as an increase in the accumulation of calprotectin possibly related to NET formation promotes coinfection between *P. aeruginosa* and *S. aureus* (Wakeman et al., 2016). Additionally, reports have been published showing that *P. aeruginosa, S. aureus*, and *B. cepacia* can cause mixed infections in the respiratory tract of patients with CF; together, lead to pulmonary exacerbations, decreased pulmonary function and destruction of the lung, so the mortality rate is higher compared to simple infections or mixed infections with two different bacteria (Zemanick et al., 2011).

CF investigations have focused on the role that bacteria on the pathogenesis of the disease, although very often other pathogens can be observed and isolated form CF patient's lungs. Of these, filamentous fungi *Aspergillus fumigatus* is the most commonly isolated (Armstead et al., 2014) and even thou detailed studies regarding its participation on CF and NET formation are scarce there are some data that could suggest an active role in the disease. *In vitro* it has been reported formation of high amounts of NETs against *A. fumigatus* hyphae compared to conidia by human neutrophils. Interestingly, NETs are unable to kill and eliminate *A. fumigatus* infection, but can reduce the growth of this fungus by trapping it into webs preventing further growth and confine the infection (McCormick et al., 2010). Additionally, other groups have related the reduction of NET formation to the presence of hydrophobin RodA, which is the major component of resting conidia surface although the precise mechanism behind this still has to be elucidated (Aimanianda et al., 2009; Bruns et al., 2010). Another fungi species that have been also isolated from CF patients are the hyaline fungi *Scedosporium apiospermum* (*Pseudollescheria boydii*) and black yeast *Exophiala dermatitidis* (Chotirmall and McElvaney, 2014) but NET formation and its importance in the disease has not been reported yet.

*Candida albicans* can also be isolated from the respiratory tract from CF patients but less frequently than other species and tend to colonize the mucoid membranes of the alimentary tract causing inflammation in the oral cavity (LiPuma, 2010; Chotirmall and McElvaney, 2014). Studies *in vitro* have shown that *C. albicans* promotes NET formation in human neutrophils and this structures can entrap and kill both the hyphae and yeast forms, (Urban et al., 2006, 2009). NET killing was mediated by neutrophil calprotectin that chelates essential ions such as Zn^2+^ and Mn^2+^ (Sohnle et al., 1996) essential for fungal growth. The last can offer a possible explanation about why this opportunistic fungus is not often found in CF patients but still more studies are needed to truly understand the mechanisms behind this.

## Concluding remarks

To date, the NETs remain a mystery and its mechanisms of formation has not been completely deciphered. Clearly, it is important for the clearance and containment of a variety of microorganisms, but equally are involved in the development immunopathology of CF, where exacerbate the disease. It is important to fully elucidate the participation of NETs in CF and its interplay with the pathogens colonizing the patient's lungs for in the future develop proper strategies to control the disease.

## Author contributions

Wrote the manuscript: SM and AS. Helped with manuscript preparation: LC, JP, and RH. Helped with commentaries and writing of the manuscript: GG.

## Funding

This work was supported by the Autonomous University of Nuevo León in México through its Support Program for Scientific and Technological Research (PAICYT) 2015. We thank to the Ethics and Research committee of the Medicine Faculty and University Hospital “Dr. José Eleuterio González” of the UANL for their help and support for the publication of this work.

### Conflict of interest statement

The authors declare that the research was conducted in the absence of any commercial or financial relationships that could be construed as a potential conflict of interest.

## References

[B1] Aimanianda V. Bayry J. Bozza S. Kniemeyer O. Perruccio K. Elluru S. R.. (2009). Surface hydrophobin prevents immune recognition of airborne fungal spores. Nature 460, 1117–1121. 10.1038/nature08264 19713928

[B2] Amulic B. Cazalet C. Hayes G. L. Metzler K. D. Zychlinsky A. (2012). Neutrophil function: from mechanisms to disease. Annu. Rev. Immunol. 30, 459–489. 10.1146/annurev-immunol-020711-074942 22224774

[B3] Armstead J. Morris J. Denning D. W. (2014). Multi-country estimate of different manifestations of aspergillosis in cystic fibrosis. PLoS ONE 9:e98502. 10.1371/journal.pone.0098502 24914809 PMC4051580

[B4] Bergsson G. Reeves E. P. McNally P. Chotirmall S. H. Greene C. M. Greally P.. (2009). LL-37 complexation with glycosaminoglycans in cystic fibrosis lungs inhibits antimicrobial activity, which can be restored by hypertonic saline. J. Immunol. 183, 543–551. 10.4049/jimmunol.0803959 19542465

[B5] Besier S. Smaczny C. Von Mallinckrodt C. Krahl A. Ackermann H. Brade V.. (2007). Prevalence and clinical significance of *Staphylococcus aureus* small-colony variants in cystic fibrosis lung disease. J. Clin. Microbiol. 45, 168–172. 10.1128/JCM.01510-06 17108072 PMC1828983

[B6] Borregaard N. Cowland J. B. (1997). Granules of the human neutrophilic polymorphonuclear leukocyte. Blood 89, 3503–3521. 9160655

[B7] Brinkmann V. Reichard U. Goosmann C. Fauler B. Uhlemann Y. Weiss D. S.. (2004). Neutrophil extracellular traps kill bacteria. Science 303, 1532–1535. 10.1126/science.1092385 15001782

[B8] Brinkmann V. Zychlinsky A. (2012). Neutrophil extracellular traps: is immunity the second function of chromatin? J. Cell Biol. 198, 773–783. 10.1083/jcb.201203170 22945932 PMC3432757

[B9] Bruns S. Kniemeyer O. Hasenberg M. Aimanianda V. Nietzsche S. Thywissen A.. (2010). Production of extracellular traps against Aspergillus fumigatus *in vitro* and in infected lung tissue is dependent on invading neutrophils and influenced by hydrophobin RodA. PLoS Pathog. 6:e1000873. 10.1371/journal.ppat.1000873 20442864 PMC2861696

[B10] Callaghan M. McClean S. (2012). Bacterial host interactions in cystic fibrosis. Curr. Opin. Microbiol. 15, 71–77. 10.1016/j.mib.2011.11.001 22137884

[B11] Chotirmall S. H. McElvaney N. G. (2014). Fungi in the cystic fibrosis lung: bystanders or pathogens? Int. J. Biochem. Cell Biol. 52, 161–173. 10.1016/j.biocel.2014.03.001 24625547

[B12] Cullen L. McClean S. (2015). Bacterial adaptation during chronic respiratory infections. Pathogens 4, 66–89. 10.3390/pathogens4010066 25738646 PMC4384073

[B13] Cutting G. R. (2015). Cystic fibrosis genetics: from molecular understanding to clinical application. HHS Public Access 16, 45–56. 10.1038/nrg3849 25404111 PMC4364438

[B14] Dubois A. V. Gauthier A. Bréa D. Varaigne F. Diot P. Gauthier F.. (2012). Influence of DNA on the activities and inhibition of neutrophil serine proteases in cystic fibrosis sputum. Am. J. Respir. Cell Mol. Biol. 47, 80–86. 10.1165/rcmb.2011-0380OC 22343221

[B15] Dwyer M. Shan Q. D'Ortona S. Maurer R. Mitchell R. Olesen H.. (2014). Cystic fibrosis sputum DNA has NETosis characteristics and neutrophil extracellular trap release is regulated by macrophage migration-inhibitory factor. J. Innate Immun. 6, 765–779. 10.1159/000363242 24862346 PMC4201867

[B16] Eisele N. A. Anderson D. M. (2011). Host defense and the airway epithelium: frontline responses that protect against bacterial invasion and pneumonia. J. Pathog. 2011:249802. 10.4061/2011/249802 22567325 PMC3335569

[B17] Filkins L. M. O'Toole G. A. (2015). Cystic fibrosis lung infections: polymicrobial, complex, and hard to treat. PLoS Pathog. 11:e1005258. 10.1371/journal.ppat.1005258 26719892 PMC4700991

[B18] Folescu T. W. da Costa C. H. Cohen R. W. F. da Conceição Neto O. C. Albano R. M. Marques E. A. (2015). Burkholderia cepacia complex: clinical course in cystic fibrosis patients. BMC Pulm. Med. 15:158. 10.1186/s12890-015-0148-2 26642758 PMC4672471

[B19] Fuchs T. A. Abed U. Goosmann C. Hurwitz R. Schulze I. Wahn V.. (2007). Novel cell death program leads to neutrophil extracellular traps. J. Cell Biol. 176, 231–241. 10.1083/jcb.200606027 17210947 PMC2063942

[B20] Gan K. H. Veeze H. J. van den Ouweland A. M. Halley D. J. Scheffer H. van der Hout A.. (1995). A cystic fibrosis mutation associated with mild lung disease. N. Engl. J. Med. 333, 95–99. 7539891 10.1056/NEJM199507133330204

[B21] Genestier A. L. Michallet M. C. Prévost G. Bellot G. Chalabreysse L. Peyrol S.. (2005). *Staphylococcus aureus* Panton-Valentine leucocidin directly targets mitochondria and induces Bax-independent apoptosis of human neutrophils. J. Clin. Invest. 115, 3117–3127. 10.1172/JCI22684 16276417 PMC1265849

[B22] Gilligan P. H. (2014). Infections in patients with cystic fibrosis: diagnostic microbiology update. Clin. Lab. Med. 34, 197–217. 10.1016/j.cll.2014.02.001 24856524 PMC7115738

[B23] Gray R. McCullagh B. McCray P. (2015). NETs and CF lung disease: current status and future prospects. Antibiotics 4, 62–75. 10.3390/antibiotics4010062 27025615 PMC4790323

[B24] Halverson T. W. R. Wilton M. Poon K. K. H. Petri B. Lewenza S. (2015). DNA is an antimicrobial component of neutrophil extracellular traps. PLoS Pathog. 11:e1004593. 10.1371/journal.ppat.1004593 25590621 PMC4295883

[B25] Hirschfeld J. Dommisch H. Skora P. Horvath G. Latz E. Hoerauf A.. (2015). Neutrophil extracellular trap formation in supragingival biofilms. Int. J. Med. Microbiol. 305, 453–463. 10.1016/j.ijmm.2015.04.002 25959370

[B26] Hubert D. Réglier-Poupet H. Sermet-Gaudelus I. Ferroni A. Le Bourgeois M. Burgel P. R.. (2013). Association between *Staphylococcus aureus* alone or combined with *Pseudomonas aeruginosa* and the clinical condition of patients with cystic fibrosis. J. Cystic Fibros. 12, 497–503. 10.1016/j.jcf.2012.12.003 23291443

[B27] Johnson L. Horsman S. R. Charron-Mazenod L. Turnbull A. L. Mulcahy H. Surette M. G.. (2013). Extracellular DNA-induced antimicrobial peptide resistance in Salmonella enterica serovar Typhimurium. BMC Microbiol. 13:115. 10.1186/1471-2180-13-115 23705831 PMC3724500

[B28] Johnson L. Mulcahy H. Kanevets U. Shi Y. Lewenza S. (2012). Surface-localized spermidine protects the *Pseudomonas aeruginosa*: outer membrane from antibiotic treatment and oxidative stress. J. Bacteriol. 194, 813–826. 10.1128/JB.05230-11 22155771 PMC3272965

[B29] Juneau R. A. Pang B. Weimer K. W. D. Armbruster C. E. Swords W. E. (2011). Nontypeable *Haemophilus influenzae* initiates formation of neutrophil extracellular traps. Infect. Immun. 79, 431–438. 10.1128/IAI.00660-10 20956567 PMC3019868

[B30] Kirchner K. K. Wagener J. S. Khan T. Z. Copenhaver S. C. Accurso F. J. (1996). Increased DNA levels in bronchoalveolar lavage fluid obtained from infants with cystic fibrosis. Am. J. Respir. Crit. Care Med. 154, 1426–1429. 10.1164/ajrccm.154.5.8912759 8912759

[B31] Klebanoff S. J. Kinsella M. G. Wightt T. N. (1993). Degradation of endothelial cell matrix heparan sulfate proteoglycan by elastase and the myeloperoxidase-H202-chloride system. Am. J. Pathol. 143, 907–917. 8395774 PMC1887211

[B32] Kolaczkowska E. Kubes P. (2013). Neutrophil recruitment and function in health and inflammation. Nat. Rev. Immunol. 13, 159–175. 10.1038/nri3399 23435331

[B33] Leshner M. Wang S. Lewis C. Zheng H. Chen X. A. Santy L.. (2012). PAD4 mediated histone hypercitrullination induces heterochromatin decondensation and chromatin unfolding to form neutrophil extracellular trap-like structures. Front. Immunol. 3:307. 10.3389/fimmu.2012.00307 23060885 PMC3463874

[B34] LiPuma J. J. (2010). The changing microbial epidemiology in cystic fibrosis. Clin. Microbiol. Rev. 23, 299–323. 10.1128/CMR.00068-09 20375354 PMC2863368

[B35] Lu T. Kobayashi S. D. Quinn M. T. DeLeo F. R. (2012). A NET outcome. Front. Immunol. 3:365 10.3389/fimmu.2012.00365 23227026 PMC3514450

[B36] Manzenreiter R. Kienberger F. Marcos V. Schilcher K. Krautgartner W. D. Obermayer A.. (2012). Ultrastructural characterization of cystic fibrosis sputum using atomic force and scanning electron microscopy. J. Cystic Fibros. 11, 84–92. 10.1016/j.jcf.2011.09.008 21996135

[B37] Marcos V. Zhou-Suckow Z. Önder Yildirim A. Bohla A. Hector A. Vitkov L.. (2015). Free DNA in cystic fibrosis airway fluids correlates with airflow obstruction. Mediators Inflamm. 2015:408935. 10.1155/2015/408935 25918476 PMC4397025

[B38] McCormick A. Heesemann L. Wagener J. Marcos V. Hartl D. Loeffler J.. (2010). NETs formed by human neutrophils inhibit growth of the pathogenic mold Aspergillus fumigatus. Microb. Infect. 12, 928–936. 10.1016/j.micinf.2010.06.009 20603224

[B39] Menestrina G. Dalla Serra M. Comai M. Coraiola M. Viero G. Werner S.. (2003). Ion channels and bacterial infection: the case of β-barrel pore-forming protein toxins of *Staphylococcus aureus*. FEBS Lett. 552, 54–60. 10.1016/S0014-5793(03)00850-0 12972152

[B40] Mulcahy H. Charron-Mazenod L. Lewenza S. (2008). Extracellular DNA chelates cations and induces antibiotic resistance in *Pseudomonas aeruginosa* biofilms. PLoS Pathog. 4:e1000213. 10.1371/journal.ppat.1000213 19023416 PMC2581603

[B41] Neeli I. Khan S. N. Radic M. (2008). Histone deimination as a response to inflammatory stimuli in neutrophils. J. Immunol. 180, 1895–1902. 10.4049/jimmunol.180.3.1895 18209087

[B42] O'Sullivan B. P. Freedman S. D. (2009). Cystic fibrosis. Lancet 373, 1891–1904. 10.1016/S0140-6736(09)60327-5 19403164

[B43] Painter R. G. Bonvillain R. W. Valentine V. G. Lombard G. A. LaPlace S. G. Nauseef W. M.. (2008). The role of chloride anion and CFTR in killing of *Pseudomonas aeruginosa* by normal and CF neutrophils. J. Leukoc. Biol. 83, 1345–1353. 10.1189/jlb.0907658 18353929 PMC2901559

[B44] Papayannopoulos V. Metzler K. D. Hakkim A. Zychlinsky A. (2010). Neutrophil elastase and myeloperoxidase regulate the formation of neutrophil extracellular traps. J. Cell Biol. 191, 677–691. 10.1083/jcb.201006052 20974816 PMC3003309

[B45] Papayannopoulos V. Staab D. Zychlinsky A. (2011). Neutrophil elastase enhances sputum solubilization in cystic fibrosis patients receiving dnase therapy. PLoS ONE 6:e28526. 10.1371/journal.pone.0028526 22174830 PMC3235130

[B46] Papayannopoulos V. Zychlinsky A. (2009). NETs: a new strategy for using old weapons. Trends Immunol. 30, 513–521. 10.1016/j.it.2009.07.011 19699684

[B47] Pilsczek F. H. Salina D. Poon K. K. Fahey C. Yipp B. G. Sibley C. D.. (2010). A novel mechanism of rapid nuclear neutrophil extracellular trap formation in response to *Staphylococcus aureus*. J. Immunol. 185, 7413–7425. 10.4049/jimmunol.1000675 21098229

[B48] Rahman S. Gadjeva M. (2014). Does NETosis contribute to the bacterial pathoadaptation in cystic fibrosis? Front. Immunol. 5:378. 10.3389/fimmu.2014.00378 25157250 PMC4127480

[B49] Ratjen F. Paul K. van Koningsbruggen S. Breitenstein S. Rietschel E. Nikolaizik W. (2005). DNA concentrations in BAL fluid of cystic fibrosis patients with early lung disease: influence of treatment with dornase alpha. Pediatr. Pulmonol. 39, 1–4. 10.1002/ppul.20134 15532079

[B50] Rochael N. C. Guimarães-Costa A. B. Nascimento M. T. C. DeSouza-Vieira T. S. Oliveira M. P. Garcia e Souza L. F.. (2015). Classical ROS-dependent and early/rapid ROS-independent release of Neutrophil Extracellular Traps triggered by Leishmania parasites. Sci. Rep. 5:18302. 10.1038/srep18302 26673780 PMC4682131

[B51] Rogan M. P. Taggart C. C. Greene C. M. Murphy P. G. O'Neill S. J. McElvaney N. G. (2004). Loss of microbicidal activity and increased formation of biofilm due to decreased lactoferrin activity in patients with cystic fibrosis. J. Infect. Dis. 190, 1245–1253. 10.1086/423821 15346334

[B52] Rowe S. M. Miller S. Sorscher E. J. (2005). Cystic fibrosis. N. Engl. J. Med. 352, 1992–2001. 10.1056/NEJMra043184 15888700

[B53] Saffarzadeh M. Juenemann C. Queisser M. A. Lochnit G. Barreto G. Galuska S. P.. (2012). Neutrophil extracellular traps directly induce epithelial and endothelial cell death: a predominant role of histones. PLoS ONE 7:e32366. 10.1371/journal.pone.0032366 22389696 PMC3289648

[B54] Sagel S. D. Sontag M. K. Accurso F. J. Bergsson G. Reeves E. P. McNally P.. (2004). Relationship between antimicrobial proteins and airway inflammation and infection in cystic fibrosis. Pediatr. Pulmonol. 44, 402–409. 10.1002/ppul.21028 19283840

[B55] Saiman L. Cacalano G. Prince A. (1990). Pseudomonas cepacia adherence to respiratory epithelial cells is enhanced by *Pseudomonas aeruginosa*. Infect. Immun. 58, 2578–2584. 1983811 10.1128/iai.58.8.2578-2584.1990 PMC258858

[B56] Sohnle P. G. Hahn B. L. Santhanagopalan V. (1996). Inhibition of *Candida albicans* growth by calprotectin in the absence of direct contact with the organisms. J. Infect. Dis. 174, 1369–1372. 10.1093/infdis/174.6.1369 8940237

[B57] Thammavongsa V. Missiakas D. Schneewind O. (2013). *Staphylococcus aureus* degrades neutrophil extracellular traps to promote immune cell death. Science 342, 863–866. 10.1126/science.1242255 24233725 PMC4026193

[B58] Urban C. F. Ermert D. Schmid M. Abu-Abed U. Goosmann C. Nacken W.. (2009). Neutrophil extracellular traps contain calprotectin, a cytosolic protein complex involved in host defense against *Candida albicans*. PLoS Pathog. 5:e1000639. 10.1371/journal.ppat.1000639 19876394 PMC2763347

[B59] Urban C. F. Reichard U. Brinkmann V. Zychlinsky A. (2006). Neutrophil extracellular traps capture and kill *Candida albicans* and hyphal forms. Cell. Microbiol. 8, 668–676. 10.1111/j.1462-5822.2005.00659.x 16548892

[B60] Voyich J. M. Braughton K. R. Sturdevant D. E. Whitney A. R. Saïd-Salim B. Porcella S. F.. (2005). Insights into mechanisms used by *Staphylococcus aureus* to avoid destruction by human neutrophils. J. Immunol. 175, 3907–3919. 10.4049/jimmunol.175.6.3907 16148137

[B61] Wakeman C. A. Moore J. L. Noto M. J. Zhang Y. Singleton M. D. Prentice B. M.. (2016). The innate immune protein calprotectin promotes *Pseudomonas aeruginosa* and *Staphylococcus aureus* interaction. Nat. Commun. 7:11951. 10.1038/ncomms11951 27301800 PMC4912628

[B62] Wang Y. Li M. Stadler S. Correll S. Li P. Wang D.. (2009). Histone hypercitrullination mediates chromatin decondensation and neutrophil extracellular trap formation. J. Cell Biol. 184, 205–213. 10.1083/jcb.200806072 19153223 PMC2654299

[B63] Welsh M. J. Smith A. E. (1993). Molecular mechanisms of CFTR chloride channel dysfunction in cystic fibrosis. Cell 73, 1251–1254. 10.1016/0092-8674(93)90353-R 7686820

[B64] Winstanley C. O'Brien S. Brockhurst M. A. (2016). *Pseudomonas aeruginosa* evolutionary adaptation and diversification in cystic fibrosis chronic lung infections. Trends Microbiol. 24, 327–337. 10.1016/j.tim.2016.01.008 26946977 PMC4854172

[B65] Yipp B. G. Kubes P. (2016). Review Article NETosis : how vital is it ? Blood 122, 2784–2795. 10.1182/blood-2013-04-457671 24009232

[B66] Young R. L. Malcolm K. C. Kret J. E. Caceres S. M. Poch K. R. Nichols D. P.. (2011). Neutrophil extracellular trap (NET)-mediated killing of *Pseudomonas aeruginosa*: evidence of acquired resistance within the CF airway, independent of CFTR. PLoS ONE 6:e23637. 10.1371/journal.pone.0023637 21909403 PMC3164657

[B67] Zawrotniak M. Rapala-Kozik M. (2013). Neutrophil extracellular traps (NETs) - formation and implications. Acta Biochim. Pol. 60, 277–284. 23819131

[B68] Zemanick E. T. Sagel S. D. Harris J. K. (2011). The airway microbiome in cystic fibrosis and implications for treatment. Curr. Opin. Pediatr. 23, 319–324. 10.1097/MOP.0b013e32834604f2 21494150

